# Management of white spot lesions by resin infiltration: a case series

**DOI:** 10.3389/froh.2026.1872881

**Published:** 2026-08-31

**Authors:** Liangyue Pang, Qinghui Zhi

**Affiliations:** 1 Hospital of Stomatology, Sun Yat-sen University, Guangzhou, Guangdong, China; 2 Guangdong Provincial Key Laboratory of Stomatology, Sun Yat-sen University, Guangzhou, Guangdong, China

**Keywords:** aesthetics, case series, management, resin infiltration, white spot lesions

## Abstract

White spot lesions (WSLs) present as color abnormalities on the tooth surface and represent early signs of demineralization beneath intact enamel, potentially affecting patients’ aesthetics and self-confidence. Management strategies for WSLs include remineralization using casein phosphopeptide–amorphous calcium phosphate (CPP-ACP) pastes or topical fluoride applications, microabrasion with an 18% hydrochloric acid and pumice mixture, and resin infiltration. Resin infiltration is a minimally invasive aesthetic treatment for WSLs, though multiple factors may influence the final aesthetic outcome. This paper reports three cases of white spot lesions treated with resin infiltration and discusses the factors that may affect treatment efficacy.

**Clinical Relevance:**

Resin infiltration is a clinically effective method for caries lesions classified as ICDAS 2–3. While white spot lesions can be treated with resin infiltration, not all cases achieve satisfactory aesthetic results. Successful implementation requires careful case selection, adherence to standardized protocols, and appropriate follow-up.

## Introduction

In white spot lesions (WSLs), the enamel layer undergoes subsurface demineralization extending from the surface layer downward, while the superficial layer remains relatively unaffected (1). Bacteria form biofilm on the enamel surface, producing a biofilm fluid undersaturated with respect to enamel mineral (1), which is transported into enamel pores (2), thus initiating the WSL. The enlargement of enamel pores by the undersaturated plaque fluid increases the water content of enamel at the expense of mineral content, altering the refractive index of translucent enamel (1). White spot lesions appear chalky due to the substantial difference in refractive indices between apatite crystals and the medium within the lesion pores, which may be either watery or air-filled (3).

Approximately 15 years ago, resin infiltration emerged as a minimally invasive technique for effectively masking enamel opacities (4). The clinical utility of this technique in the treatment of white spot lesions has since been validated by a wealth of published evidence, including expert consensus statements (5–9). The low-viscosity resin infiltrant penetrates the porosity of carious enamel, and its refractive index—higher than that of water and air (10)—improves the aesthetic appearance of teeth through a camouflage effect. Through three clinical cases, this case report explores the indications and factors influencing the efficacy of resin infiltration in treating enamel white spot lesions.

## Case 1

### Clinical presentation

**Figure 1:**
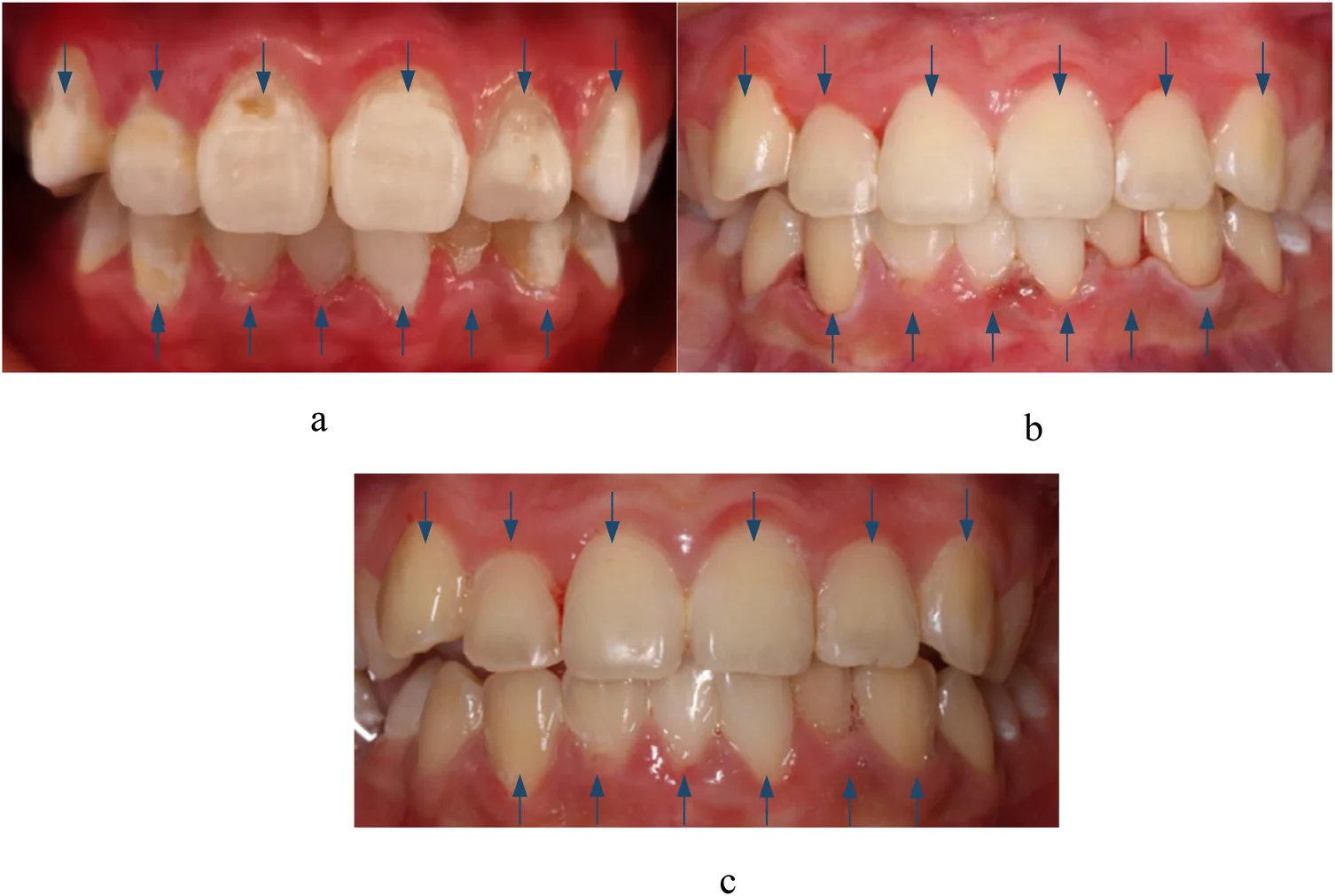
Case 1, a fourteen-year female patient treated with resin infiltration for white spots on the upper anterior. **(a)** Preoperative view; **(b)** Immediate postoperative result after resin infiltration; **(c)** 3-months follow up. Arrows indicate the lesions of interest.

A 14-year-old female patient with no systemic disease presented to our clinic concerned about irregular chalky white and brown spots on her teeth that significantly impacted her appearance (Figure 1a). According to the International Caries Detection and Assessment System (ICDAS) II criteria (11), WSLs present on the labial surfaces of teeth 13 and 11 were classified as ICDAS 3, while those on teeth 12, 21, 22, 23, and 33–43 were classified as ICDAS 2. All white spot lesions were considered active, defined as whitish/yellowish, opaque, dull surfaces that felt rough on probing (11). The patient's oral hygiene was poor, with gingival redness and swelling. Prior to resin infiltration treatment, the patient underwent supragingival scaling and received oral hygiene instructions. The resin infiltration procedure was performed one week later.

## Treatment procedure

A non-invasive resin infiltration technique was chosen to treat these WSLs. Oral rubber dam isolation was employed, and a gingival barrier agent was used to seal around the rubber dam to protect the gingiva from hydrochloric acid corrosion. The resin infiltration procedure was performed in accordance with the manufacturer's guidelines. The WSLs were first treated with micro-abrasion, followed by etching with Icon-Etch for 2 min. After rinsing and drying the enamel surfaces, Icon-Dry was applied for 30 s to eliminate any moisture trapped in the microporosities. The lesions were then infiltrated with Icon-Infiltrant and light-cured for 40-second intervals (Icon, DMG, Hamburg, Germany). Subsequently, Icon-Infiltrant was applied for a second time and light-cured for 40 s. Finally, the rough surface was smoothed using a silicone rubber polishing point.

## Treatment outcome

Immediately after treatment, the white spot lesions showed noticeable aesthetic improvement. In the distal middle region of tooth 21, a demineralization area was initially treated with the infiltration resin technique but failed; this area was subsequently treated with an aesthetic resin filling. The patient was highly satisfied with the aesthetic outcome, and photographs were taken to document the results (Figure 1b). The patient was advised on proper oral hygiene practices and instructed to brush daily with fluoride toothpaste for the next 3 months.

At the 3-month follow-up, clinical examination and photographs (Figure 1c) revealed no progression of the early carious lesions. No new stains were noted on teeth 13–23 and 33–43, and the color and brightness achieved by resin infiltration were maintained.

## Case 2

### Clinical presentation

**Figure 2:**
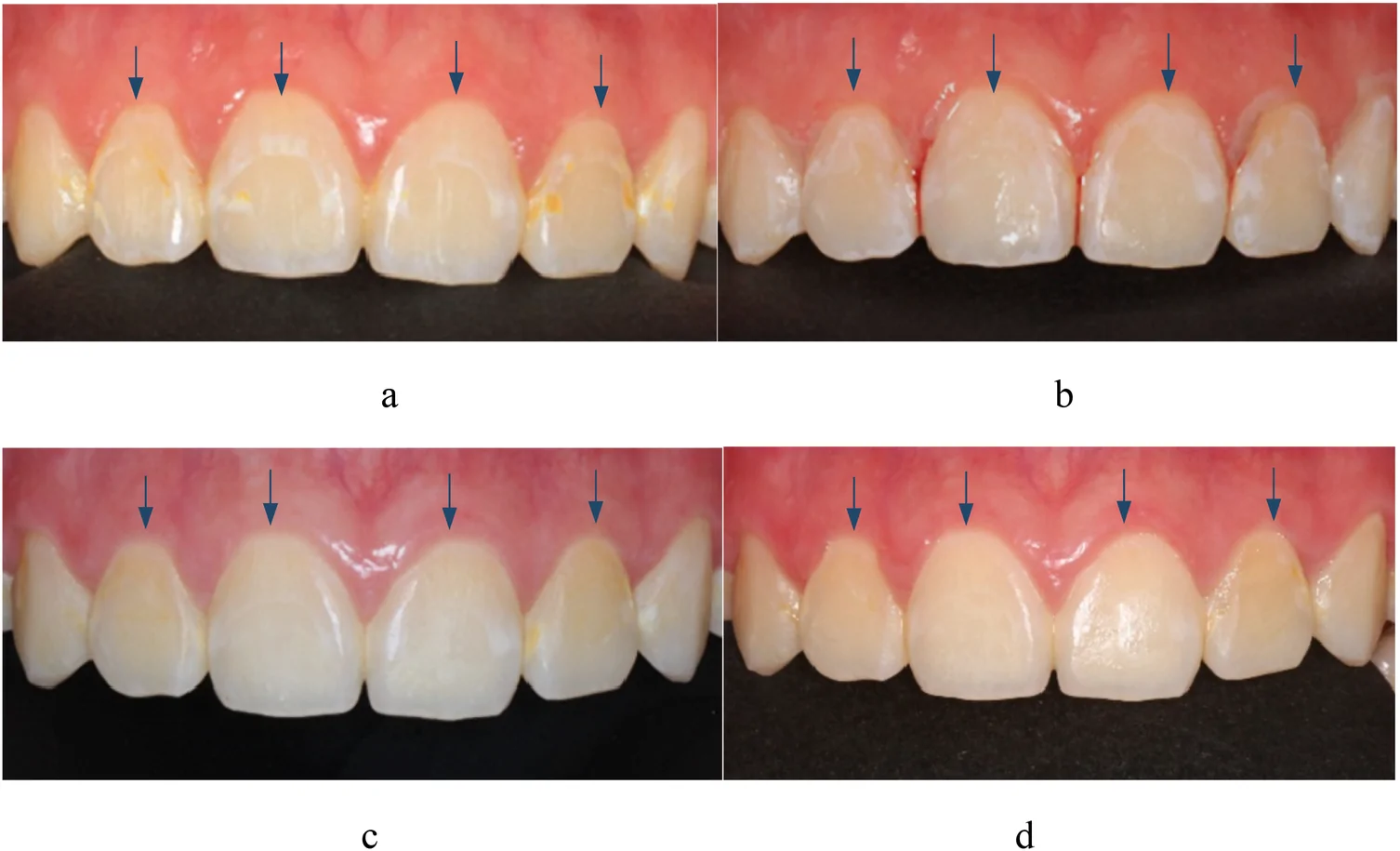
Case 2—treatment of post-orthodontic white spots with resin infiltration. **(a)** Baseline frontal view of teeth 12, 11, 21, 22; **(b)** Clinical appearance immediately after resin infiltration; **(c)** 6-month follow-up; **(d)** 24-month follow-up. Arrows indicate the lesions of interest.

A 14-year-old girl who had recently completed orthodontic treatment presented to our clinic concerned about chalky discoloration on her upper front teeth affecting their aesthetics. White spot lesions were observed on all six upper anterior teeth from tooth #12 to tooth #22. The enamel surface appeared whitish and shiny. It felt hard and smooth when the tip of the probe was moved across it, and the caries lesions were typically located at some distance from the gingival margin. According to ICDAS II criteria (11), these lesions were classified as ICDAS 2 (Figure 2a) and were inactive. The patient demonstrated average oral hygiene, with no visible signs of gingival redness or swelling.

## Treatment procedure

Resin infiltration was also used to treat these WSLs. Oral rubber dam isolation was employed, and a gingival barrier agent was used to seal around the rubber dam to protect the gingiva from hydrochloric acid corrosion. The resin infiltration procedure was performed in accordance with the manufacturer's guidelines. The WSLs on teeth #12 to #22 were etched with Icon-Etch for 2 min without microabrasion. After rinsing and drying the enamel surfaces, Icon-Dry was applied for 30 s to eliminate any moisture trapped in the microporosities. The lesions were then infiltrated with Icon-Infiltrant and light-cured for 40-second intervals (Icon, DMG, Hamburg, Germany). Subsequently, Icon-Infiltrant was applied for a second time and light-cured for 40 s. Finally, the rough surface was smoothed using a silicone rubber polishing point.

## Treatment outcome

Following resin infiltration, although there was a reduction in WSLs, the improvement was not immediately apparent (Figure 2b). The patient received oral hygiene education and was scheduled for a 6-month follow-up (Figure 2c). Remarkably, after 6 months, there was noticeable improvement in the WSLs of the patient's upper anterior teeth. With each subsequent observation, the improvement became more pronounced, and by the 24-month mark, the WSLs had nearly disappeared (Figure 2d).

## Case 3

### Clinical presentation

**Figure 3:**
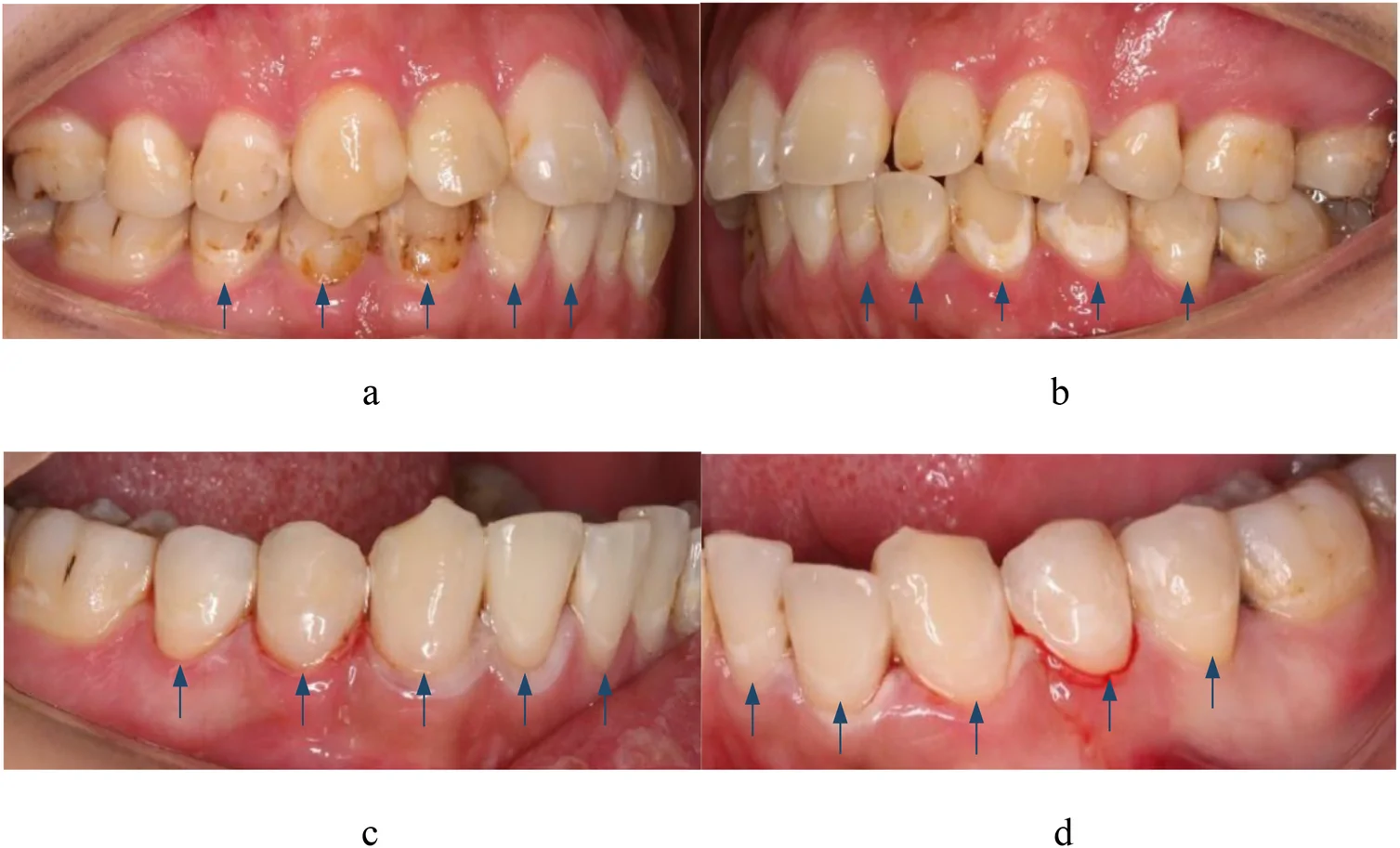
Case 3—treatment of white spot lesions and secondary caries in mandibular teeth with resin infiltration and aesthetic resin filling technique. **(a,b)** White spot lesions and secondary caries in mandibular teeth before treatment; **(c,d)** Immediate view after treatment with resin infiltration and aesthetic resin filling technique. Arrows indicate the lesions of interest.

A 15-year-old male patient, recently discharged from orthodontic treatment, presented at the clinic concerned about perceived decayed cavities in his mandibular dentition and requested restorative dental treatment. Upon examination, the patient exhibited various dental anomalies and requested treatment for teeth #32–#35 and #42–#45. Specifically, teeth 46 and 45 displayed chalky coloration with yellowish-brown discoloration. Teeth 44 and 43 presented with fillings accompanied by yellowish-brown changes. Tooth 42 exhibited yellowish-brown alterations in the proximal middle region and chalky changes in the cervical area. Teeth 41, 31, and 32 displayed chalky changes in their cervical regions. Notably, significant enamel demineralization accompanied by slight pigmentation was observed in the cervical areas of teeth 33 and 34. Similarly, teeth 35 and 36 exhibited minor enamel demineralization in the proximal middle region with slight pigmentation (Figures 3a,b). Based on ICDAS criteria, the enamel alterations observed in teeth 45, 44, 43, and the proximal area of 42 were assigned an ICDAS score of 4 and classified as active lesions. The enamel changes observed in teeth 46, 41, 31, 32, 33, 34, 35, and 36 were assigned an ICDAS score of 3 and classified as active lesions.

## Treatment procedure

Following consultation with the patient, a comprehensive treatment plan was formulated. This plan included restoration of teeth 45, 44, 43, and the proximal mesial aspect of tooth 42 using aesthetic resin filling technique. Additionally, the cervical regions of teeth 42, 32, 33, 34, and 35 were treated with infiltration resin technique. Pigmentation removal from teeth 46 and 36 was addressed using polishing technique exclusively, supplemented with tooth brushing using fluoridated toothpaste.

Oral rubber dam isolation was employed, and a gingival barrier agent was used to seal around the rubber dam to protect the gingiva from corrosion. The cervical regions of teeth 42, 32, 33, 34, and 35 were etched with Icon-Etch for 2 min. After rinsing and drying the enamel surfaces, Icon-Dry was applied for 30 s to eliminate any moisture trapped in the microporosities. The lesions were then infiltrated with Icon-Infiltrant and light-cured for 40-second intervals (Icon, DMG, Hamburg, Germany). Subsequently, Icon-Infiltrant was applied for a second time and light-cured for 40 s. Teeth 45, 44, 43, and the mesial proximal surface of tooth 42 were restored using the Master Resin aesthetic composite resin technique. First, the carious tooth tissue was removed, followed by selective etching with 37% phosphoric acid. After rinsing and drying, an eighth-generation bonding agent was applied, followed by layered aesthetic composite resin restoration using Master Resin. Finally, the rough surfaces were smoothed using a silicone rubber polishing point, and the pigmentation of teeth 46 and 36 was addressed.

## Treatment outcome

Upon completion of the treatment regimen, there was marked enhancement in the aesthetic appearance of the patient's mandibular dentition, resulting in a high level of patient satisfaction (Figures 3c,d). This case is ongoing follow-up.

## Discussion

Several methods have been attempted to manage WSLs and contain caries lesion progression (9, 20). The first category of therapies is primarily directed toward lesion activity control and remineralization, such as fluoride and CPP-ACP. Although fluoride plays a crucial role in the prevention and management of dental caries, this approach does not yield immediate results and requires consistent maintenance through proper oral hygiene practices. Additionally, it is not always aesthetically successful (5, 12). Another study suggests that applying CPP-ACP for 3 months, followed by the use of fluoride toothpaste for the subsequent 3 months, contributes to the regression of post-orthodontic white spot lesions (10).

The second category focuses predominantly on aesthetic masking and optical modification, including microabrasion and resin infiltration. For instance, microabrasion with 18% hydrochloric acid (HCl) and polishing with pumice powder can improve the aesthetic effect primarily through physical removal of discolored enamel. Although this technique removes up to 250 microns of enamel and is more conservative than traditional resin composite restoration, it remains invasive due to enamel removal (13). Recent studies have indicated that resin infiltration is a more effective treatment for WSLs (14). Therefore, in these clinical cases, resin infiltration was selected for its ability to fill, reinforce, and stabilize demineralized enamel without drilling or compromising healthy tooth structure. By penetrating the porous lesion body, resin infiltration forms a diffusion barrier that prevents further acid penetration and ion loss, arresting lesion progression while immediately improving aesthetic appearance. Accordingly, although this report does not propose a novel method, its technical contribution lies in illustrating the clinical application and procedure-related considerations of an established resin infiltration technique across white spot lesions with different clinical presentations and ICDAS classifications.

No consensus exists on the objective criteria defining resin infiltration success or failure. In this report, treatment was considered successful if acceptable immediate aesthetics (marked reduction or disappearance of the white spot) were achieved, or if lesion progression was halted or clearly improved during follow-up. Conversely, treatment was regarded as unsuccessful when aesthetic improvement fell short or when lesion progression was observed either radiographically or clinically.

The three cases of white spot lesions described in this report are primarily attributable to variations in the refractive index (RI) within this porous region compared to other areas of the enamel. The increased porosity of the enamel, along with enlarged crystalline spaces, permits the infiltration of watery medium (RI: 1.33) or air (RI: 1.0) into spaces that would typically be occupied by apatite crystals (RI: 1.62) (4). The higher the difference in RI, the more visible light is scattered between the crystals and the more whitish the lesion appears (3). Icon (RI: 1.46) is a photopolymerizable resin characterized by low viscosity and a high penetration coefficient. Due to its properties, the material can effectively infiltrate the porous structure of carious enamel through capillary action (10). When the pores are infiltrated with Icon, light scattering is substantially diminished due to the minimal RI difference between the resin and the apatite crystal pores, rendering the lesion visually comparable to sound enamel (3), thereby enhancing the aesthetic appearance of the teeth through a camouflage effect (5, 12). Some evidence indicates that resin infiltration treatment can effectively prevent lesion progression of WSLs in anterior teeth for at least 8 years (8).

According to these clinical cases, the efficacy of resin infiltration is contingent upon multiple factors. Primarily, in terms of case selection, the findings of this limited case series suggest that resin infiltration may offer greater efficacy for lesions classified as ICDAS 2–3, although this observation warrants confirmation in larger prospective studies. ICDAS is a clinical scoring system used to detect and assess dental caries (11). ICDAS 2 is defined as distinct enamel opacity detected without air drying. ICDAS 3 represents the early breakdown of enamel due to caries without visible dentin exposure, while ICDAS 4 refers to a dark shadow from the underlying dentin, with or without enamel breakdown (11). *In vitro* and *in vivo* studies have confirmed that resin infiltration can be used to treat lesions categorized as ICDAS code 3 (15, 16), and Cases 1 and 3 also confirm this finding. In contrast, ICDAS 4 refers to a dark shadow originating from deep dentin, with or without enamel surface breakdown (11). In such lesions, because the shadow arises from deep dentin, resin infiltration cannot repair the underlying dentinal destruction; consequently, the use of resin infiltration alone often fails to achieve satisfactory aesthetic outcomes. Moreover, even if the enamel layer undergoes infiltration, this does not halt the progression of the dentinal lesion; instead, it may mask the underlying pathology, allowing the disease to progress undetected. ICDAS 5 presents as a distinct cavitation with clear dentin exposure, where resin infiltration alone is insufficient to restore the lost tooth structure, and direct restorative treatment such as composite resin filling is required, as demonstrated in Case 3. Therefore, in clinical decision-making, the indications for resin infiltration should be strictly observed, and its application to deep lesions corresponding to ICDAS 4–5 should be avoided.

The findings of the three cases also indicate that even for carious white spots classified as ICDAS 2, resin infiltration techniques do not invariably yield successful outcomes. One contributing factor may be the omission of microabrasion (16). For example, in Case 2, the absence of microabrasion during the resin infiltration process resulted in inadequate resin penetration and unsatisfactory outcomes. Another potential contributing factor may be the insufficient duration of resin infiltration application. It has been shown that increased infiltration time (up to 30 min) can improve the aesthetic outcome of resin infiltration (17, 18). Nevertheless, over time, the white spots gradually diminished. We hypothesize that this phenomenon may be due to marginal remineralization of demineralized enamel around the infiltrated resin (19). However, we have no quantitative data (e.g., laser fluorescence) to support this, so this explanation remains preliminary. In addition, the marked improvement in gingival health and oral hygiene in Case 2 could affect the aesthetic appearance of the lesion independently of the resin infiltration itself. This is an important confounder, and caution is needed when interpreting the aesthetic results.

Other factors may also contribute to the final aesthetic outcome of resin infiltration, including improved plaque control during follow-up, reduced gingival inflammation, changes in enamel hydration and its effect on optical appearance over time, and natural lesion stabilization. For instance, this may include passive remineralization or altered light scattering properties on the lesion surface under reduced demineralization conditions. Therefore, it is premature to consider this treatment a failure when the effectiveness of resin infiltration appears limited. Long-term monitoring of cases and providing appropriate oral health guidance to patients is recommended. Additionally, appropriate oral hygiene practices were also recommended to optimize gingival health prior to the resin infiltration treatment.

It should be noted that although Case 3 achieved favorable aesthetic outcomes, the final improvement cannot be attributed solely to resin infiltration, as some teeth were treated with both resin infiltration and composite resin restorations. Furthermore, the immediate post-operative aesthetic appearance may have been partially influenced by enamel dehydration and transient optical effects induced by the materials and procedures, and thus may not reflect the long-term stable outcome. In addition, the overall improvement in oral hygiene, plaque control, and gingival health during the treatment period could also act as confounding factors affecting the long-term visual appearance of the lesions. In this case, the patient declined radiographic examination. Consequently, radiographic documentation and long-term longitudinal follow-up data were lacking, and the present findings represent only short-term results. Given these considerations, a more cautious interpretation of the final outcomes is warranted when resin infiltration is combined with other therapeutic modalities.

The case reports have several limitations. First, the clinical photographs were not captured under standardized conditions. Variations in lighting, focus, angulation, contrast, and image exposure were observed across cases. Specifically, the baseline image of Case 1 was taken with a cell phone and lacks adequate sharpness, which limits detailed evaluation of the initial lesion characteristics and may affect the reliability of visual comparisons between baseline and follow-up outcomes. Second, the follow-up schedules varied considerably among the three cases: Case 1 was followed for 3 months, Case 2 for 24 months, and Case 3 had no post-treatment follow-up. This heterogeneity hinders meaningful comparison across cases and inevitably narrows the scope of our conclusions. Third, the sample size is small (three cases), and the findings may not be generalizable to broader patient populations. Future studies with standardized photographic protocols, objective assessment tools, and larger cohorts are needed to validate these observations.

## Conclusion

This case series illustrates that resin infiltration may be a useful minimally invasive option for selected white spot lesions, particularly when appropriate case selection, standardized clinical procedures, and follow-up are performed. However, the outcome of this technique can be affected by many factors, such as technique sensitivity and depth of caries progression. Therefore, successful implementation requires careful selection of appropriate cases, adherence to standardized procedures, and appropriate follow-up.

## Data Availability

The original contributions presented in the study are included in the article/Supplementary Material, further inquiries can be directed to the corresponding author.

## References

[1] Thylstrup A Bruun C Holmen L. *In vivo* caries models–mechanisms for caries initiation and arrestment. Adv Dent Res. (1994) 8(2):144–57. 10.1177/08959374940080020401 7865069

[2] Shellis RP. A scanning electron-microscopic study of solubility variations in human enamel and dentine. Arch Oral Biol. (1996) 41(5):473–84. 10.1016/0003-9969(96)00140-9 8809311

[3] Paris S Meyer-Lueckel H. The potential for resin infiltration technique in dental practice. Dent Update. (2012) 39(9):623–6, 628. 10.12968/denu.2012.39.9.623 23479851

[4] Paris S Meyer-Lueckel H. Masking of labial enamel white spot lesions by resin infiltration—a clinical report. Quintessence Int (Berl). (2009) 40(9):713–8. PMID: 19862396. 19862396

[5] Tirlet G Chabouis HF Attal J-P. Infiltration, a new therapy for masking enamel white spots: a 19-month follow-up case series. Eur J Esthet Dent. (2013) 8(2):180–90. PMID: 23712339. 23712339

[6] Ståhl L Papageorgiou SN Westerlund A Naoumova J. Outcomes of resin infiltration for white spot lesions at different time points: a systematic review and meta-analysis. Eur J Orthod. (2026) 48(3):cjag021. 10.1093/ejo/cjag021 42139649 PMC13178833

[7] Xia L Zhou C Mei P Jin Z He H Wang L. Expert consensus on the prevention and treatment of enamel demineralization in orthodontic treatment. Int J Oral Sci. (2025) 17(1):13. 10.1038/s41368-024-00335-7 40021614 PMC11871012

[8] Omoto ÉM Oliveira LC Rocha RS Machado LS Bresciani E Prakki A. An 8-year follow-up of resin infiltration on anterior white spot lesions. J Indian Soc Pedod Prev Dent. (2023) 41(1):83–5. 10.4103/jisppd.jisppd_136_22 37282417

[9] Alrebdi AB Alyahya Y. Microabrasion plus resin infiltration in masking white spot lesions. Eur Rev Med Pharmacol Sci. (2022) 26(2):456–61. 10.26355/eurrev_202201_27870 35113421

[10] Ferreira DAH Aires CP Figueiredo RCBQD Sousa FB. High amount of organic matter during caries formation reduces remineralization and resin infiltration of enamel caries. Caries Res. (2018) 52(6):580–7. 10.1159/000488211 29723862

[11] Dikmen B. ICDAS II criteria (International Caries Detection and Assessment System). J Istanbul Univ Fac Dent. (2015) 49(3):63–72. 10.17096/jiufd.38691 PMC5573507 28955548

[12] Attal J-P Atlan A Denis M Vennat E Tirlet G. White spots on enamel: treatment protocol by superficial or deep infiltration (part 2). Int Orthod(2014) 12(1):1–31. 10.1016/j.ortho.2013.12.011 24503373

[13] Simon LS Dash JK U D Philip S Sarangi S. Management of post orthodontic white spot lesions using resin infiltration and CPP-ACP materials- a clinical study. J Clin Pediatr Dent. (2022) 46(1):70–4. 10.17796/1053-4625-46.1.12 35311975

[14] Torres CRG Borges AB. Color masking of developmental enamel defects: a case series. Oper Dent. (2015) 40(1):25–33. 10.2341/13-346-T 25136905

[15] Paris S Bitter K Naumann M Dörfer CE Meyer-Lueckel H. Resin infiltration of proximal caries lesions differing in ICDAS codes. Eur J Oral Sci. (2011) 119(2):182–6. 10.1111/j.1600-0722.2011.00807.x 21410560

[16] Tiuraniemi S Yli-Mannila J Havela P Käkilehto T Vähänikkilä H Laitala ML. Success of resin infiltration treatment on interproximal tooth surfaces in young adults-A practice-based follow-up study. Clin Exp Dent Res. (2021) 7(2):189–95. 10.1002/cre2.349 33242226 PMC8019756

[17] Marouane O Manton DJ. The influence of lesion characteristics on application time of an infiltrate applied to MIH lesions on anterior teeth: an exploratory *in vivo* pilot study. J Dent. (2021) 115:103814. 10.1016/j.jdent.2021.103814 34543698

[18] De Carvalho GG Pires AC De Sousa FB. Influence of infiltrant application time on the reduction of opaqueness of proximal enamel caries. Indian J Dent Res. (2019) 30(1):52–6. 10.4103/ijdr.IJDR_267_17 30900657

[19] Zhou Y Wang Q Cui T Li J Lo ECM Hao G. Comparative assessment of four treatments for post orthodontic white spot lesions over a 24-month period: a randomized controlled clinical trial. BMC Oral Health. (2026 May 30) 26:8740. 10.1186/s12903-026-08740-6 PMC13483708 42218470

[20] Oliveira A Felinto L Francisconi-dos-Rios L Moi G Nahsan F. Dental bleaching, microabrasion, and resin infiltration: case report of minimally invasive treatment of enamel hypoplasia. Int J Prosthodont. (2020) 33(1):105–10. 10.11607/ijp.6232 31860920

